# “All I know is that a disabled person is someone who is crippled”: Using narratives of parents to unmask the misconceptions of mild intellectual disabilities concept as a learning support hindrance

**DOI:** 10.1177/17446295241242573

**Published:** 2024-03-26

**Authors:** Nancy Phyllis Makhosazane Mabaso

**Affiliations:** Ringgold: 61799 University of Johannesburg, Pimville, South Africa

**Keywords:** home-based learning support, learning support hindrance, mild intellectual disabilities, misconceptions, parental empowerment

## Abstract

Parental involvement in providing learning support plays a crucial role in children's academic achievement. However, this support is often constrained when children are diagnosed with mild intellectual disabilities and require additional assistance. This study aimed to explore the experiences of parents in supporting children with such diagnoses. Adopting a phenomenological design within an interpretive paradigm and qualitative approach, data were collected from 23 purposefully selected parents of learners with mild intellectual disabilities attending three inclusive schools in disadvantaged areas of Gauteng, South Africa. Semi-structured interviews were used, and the data were analyzed through thematic content analysis, with Bronfenbrenner's bio-ecological systems theory serving as the framework. The results indicated that parents' misunderstanding of mild intellectual disability posed a significant barrier to effective learning support. The study concluded that empowering parents through psycho-education is essential, highlighting the need for adjustments in policy and practice.

## Introduction

In this article, the author asserts that parents whose children are diagnosed with mild intellectual disabilities face challenges in providing learning support, due to misconceptions of the intellectual disability concept and limited knowledge of techniques to support their children. The article focuses on parents’ misconceptions out of interest in establishing the South African educational system redress progress since the promulgation of the Education White Paper 6 policy on inclusive education, which recommended parental involvement in learning support provision (DoE, 2014). The limited literature in the field asserts that misconceptions about intellectual disabilities pose learning support hindrances (Baker et al. 2021). In addition to misconceptions, global literature postulates that the intellectual disability concept in general attracts negative connotations and stigma (Pelleboer-Gunnink and Embregts 2021). According to APA (2013), an intellectual disability (ID) is classified as a neurodevelopmental disorder consisting of limitations in adaptive functioning due to a low intelligence quotient (IQ), a quantitative representation of an individual’s score on a standardized intelligence test. The DSM 5-TR classifies intellectual disability into four categories based on the IQ range. The intellectual disability categories range from mild intellectual disability within the IQ range of 52-69, Moderate intellectual disability within the 36-51 IQ range, Severe intellectual disability within the 20-35 IQ range, and Profound intellectual disability below 19 (APA, 2022). McKenzie (2021) asserts that each category presents with certain characteristics, which manifest differently in individuals living with the disability. Intellectual disability diagnosis can be made through the administration of an IQ assessment tool by relevant professionals such as psychometrists and or educational psychologists (Landsberg, Kruger and Swart 2019). The diagnosis is made when the deficits affect the cognitive, conceptual, and social domains. Furthermore, the challenges are observed in the learners’ reasoning; problem-solving; planning; abstract thinking; judgment; academic learning; ability to learn a new skill; ability to function independently, and experiential abilities, which adversely affect the child’s educational performance, depending on the category (Aziz et al. 2016; DoH, UK, 2001). Children with intellectual disabilities have a limited ability to learn without remedial support, which ought to be facilitated by educators in the educational setting, and by parents in the home setting (Baker et al., 2021; Shree and Shukla 2016). This paper focused on the mild intellectual disability category as these children present with a higher adaptive functioning ability and are often placed in inclusive schools due to the scholastic challenges they experience in mainstream schools. The term parents in this paper, and the South African context refers to either biological parents or caregivers of children diagnosed with mild intellectual disability (Sibanda 2021). According to APA (2022), there may be no obvious difficulties for preschool children with mild intellectual disabilities. The challenges are identified once these children reach their school-going age and enter the formal education system. However, Brown (2013) states that the reports on the onset of the symptoms are often encountered with resistance, given that parents are alerted for the first time about the intellectual disability.

### Conceptualizing the educational needs of children who live with mild intellectual disabilities

Intellectual disabilities pose a demand for additional support with children’s day-to-day functioning in both the school and the home contexts (Department of Health, 2001: 14). This suggests that parental involvement in learning support facilitation is necessary for the home context, alongside the support provided by the teachers in the school context. Hamlin and Flessa (2018) assert that parental involvement in providing learning support is key to the educational success of their children, and more so for children with mild intellectual disabilities as they require more support compared to their typically developing peers (DoE 2014; Engelbrecht and Green 2009; Garrotte, 2017). However, the process of providing learning support is hindered if parents lack clarity about the disability and the educational needs of their children with mild intellectual disabilities (Engelbrecht and Green 2009).

There is a dearth of research on the lived experiences of parents in providing home-based learning support to children with mild intellectual disabilities globally because these children appear to ‘typically’ develop and are often undiagnosed (Burrack et al., 2011b). Unlike in moderate, severe, and profound severity levels of intellectual disabilities as listed APA (2022), children with mild intellectual disabilities can achieve a relatively independent living, with appropriate support. Hamlin and Flessa (2018) further accentuate that it is therefore very common to miss intellectual disabilities in school-going children as these children often function adequately in non-school related tasks. Additionally, McKenzie (2021) asserts that parents of children with intellectual disabilities tend to naturally lean more toward highlighting what their children can do outside the classroom environment. Moreover, parents often use these non-school-related abilities to dismiss the possibility of intellectual disabilities being present in their children. Such findings confirm the existing gaps in parents’ knowledge of intellectual disabilities. The lack of research on whether parents know about, and how to provide learning support to learners with intellectual disabilities is also consistently highlighted as gaps in the literature (Maddock and Maroun, 2018). Thus, this study sought to investigate whether a relationship exists between parents’ misconceptions about mild intellectual disabilities and the effective learning support provision within the home environment, using the South African context.

### South African educational milestones to support learners with intellectual disabilities

Post attaining the democracy milestone in 1994, South Africa called for the reformation of the education system to enhance accessibility to education for all citizens, regardless of their abilities. One of the ways to set the reformation in motion was the adoption of the Education White Paper 6 policy document on inclusive education (DoE, 2001). Inclusive education suggested the adaptation of teaching and learning practices to complement the Bill of Rights as laid out in the South African Constitution of 1996, which advocated access to education for all citizens, including those with intellectual disabilities (DoE, 2014). In addition, White Paper 6 on the inclusive education system cited parents as the key stakeholders and role-players in the scholastic success of their children Magano and Ramnarain (2015). Available South African literature concentrates on the effects of intellectual disabilities on the academic potential of the learners and seldom explores the effects of intellectual disabilities on the parents, who have a responsibility to provide home-based learning support to their children (Pasha, 2012; Garrote, 2017).

In support of the inclusive education system implementation in South Africa, the Department of Basic Education (DBE) (DBE, 2020) introduced a new policy in 2014 called the Screening, Identification, Assessment and Support (SIAS). The SIAS policy document emphasized the importance of identifying the learners at risk for intellectual disabilities through the completion of special needs analysis (SNA) forms, as part of the screening procedures conducted during the Foundation Phase (DBE, 2015). The completion of the SNA forms would require parents to share background developmental milestone information of their children from early childhood, and focus would be placed on whether there were any delays in milestone attainment (DBE, 2015). Sharing of such information calls for parents to be willing and able to play a supportive role in the education of their children (Ntekane, 2018). Literature reveals that although the reasons are not clear, challenges faced by parents in providing adequate learning support to their children with mild intellectual disabilities continue to surface even after the introduction and implementation of an inclusive education system (Maddock and Maroun, 2018). Moreover, research indicates that the lack of empowerment of the key stakeholders such as parents, impedes the drive towards a thriving inclusive education system (Donald et al., 2014). In addition, although parents are willing to support their children with mild intellectual disabilities, they face more challenges in providing adequate learning support (DoE, 2015). Thus, this study explored the experiences of parents in supporting their children diagnosed with mild intellectual disabilities in inclusive schools, in the Gauteng province, South Africa, to bridge the gap in literature through unmasking the actual challenges faced when providing home-based learning support.

## Theoretical framework

Bronfenbrenner’s bio-ecological systems (1979) underpinned and located this study within the existing theoretical framework (Henning, Van Rensburg and Smith 2004: 25). According to Sefotho (2018), the theoretical framework refers to the theory that a researcher chooses to guide their research, thereby making assumptions about their worldview (Hennink et al., 2020). Bronfenbrenner’s theory is an example of a multi-dimensional, contextualist theory of human development (Bronfenbrenner, 2005 in Ceci, 2006). In addition, Bronfenbrenner contends that to better understand human development, it is important to study the individual’s entire ecological system (Elliot and Tudge, 2007). The general assumption that most parents struggle with learning support provision motivated the researcher to learn about the context in terms of the various systems in which these parents exist.

In his bio-ecological system theory, Bronfenbrenner postulates that children’s growth and development are influenced by direct and indirect interactions with their multi-layered context (Bronfenbrenner, 2005). Ceci (2006) refers to the multi-layers as systems, namely the microsystem (the immediate environment), mesosystem (the relationship between two or more microsystems), exosystem (the environment in which the learner is not directly involved), macrosystem (the most distal level of the environment such as economic structures) and chronosystem (the interactions between systems over time). The morpheme ‘bio’ to the term ecological accentuates Bronfenbrenner’s long-held view that personal characteristics remain crucial through interactions with the layers of the system as these determine how the person interprets and responds to the events through the interaction (Bronfenbrenner and Morris, 2006; Ceci, 2006; Tudge et al., 2009).

This study located itself within the immediate environment (microsystem) of Bronfenbrenner’s bio-ecological systems framework and sought to explore the learning support interaction between parents and their children within the home setting. Moreover, the study aimed to establish what the learning support hindrances are within the immediate home environment (microsystem) of children diagnosed with mild intellectual disabilities.

## Methodology

This study employed an interpretive paradigm and a phenomenological design, within the qualitative research approach to give an insider’s perspective of experiences (Leedy and Ormond, 2010; Samuels, 2019). The interpretative paradigm enabled the description of the participants’ experiences, in this case, the parents whose children are diagnosed with mild intellectual disabilities as perceived in their real-life situation (Creswell, 2009, 2014). In addition, the interpretive paradigm accommodated multiple realities of the participants’ worldviews as they gave insights into their experiences (Sefotho, 2018).

The phenomenological design considers the experience of being human in all its various aspects, but especially in terms of the things that matter to them, and which constitute their lived world, and regards perceptions as the subjective source of information (Samuels, 2019; Shudak, 2018). This design was, therefore, suitable for the study as it granted the researcher access to what really matters to parents as they navigate learning support provision to their children with mild intellectual disabilities. Furthermore, this study being embedded within a qualitative research approach enabled an in-depth empathetic understanding of the finer details of parents’ experiences during individual interviews (Kalman, 2019). According to Showkat (2017), employing a qualitative research approach also provides rich data from the participants as they share their lived experiences. Thus, in this study, parents shared their lived experiences as well as the specific challenges they face when supporting their children with mild intellectual disabilities in completing their school tasks.

### Sampling

As alluded to by Samuels (2019), the purposive sampling method was employed in the study to do an in-depth investigation on a small number of parents whose children had been assessed and diagnosed with mild intellectual disabilities by an educational psychologist. Twenty-three (17 females and 6 males) parents from 3 different inclusive schools in Gauteng, South Africa participated in the study through in-person semi-structured interviews. Notwithstanding Hesse-Bibber and Leavy’s (2011) statement that in purposive sampling, generalizing data beyond the sample group is inappropriate, the participants represented the diverse South African population and provided rich data, which according to Guest et al. (2013) and Adams et al. (2014) can be applicable globally. Another common trait possessed by the participants is that they all came from impoverished backgrounds. Sweetman (2022) postulates that in addition to other challenges, impoverished backgrounds are characterized by challenges in the educational system such as limited communication between the schools and parents on issues pertaining to learning support. The reason for sampling participants from impoverished backgrounds was to elicit this study data from participants who are part of communities that are characterized by educational challenges. Additionally, three inclusive schools from different areas were chosen to establish whether race and culture would present any similarities or noticeable differences to the phenomenon being researched. In terms of race, the first inclusive school was predominantly Black, inclusive school two was predominantly Indian, and the third inclusive school was predominantly White. Tables 1,2 & 3 below depict the demographic information of the 23 participants.

**Table 1.** Participants’ profile: Inclusive school 1.

Participant (Pseudonym)	Age	Gender	Occupation
Parent 1 (Amelia)	39	Female	Chef
Parent 2 (Ayanda)	51	Female	Unemployed
Parent 3 (Andile)	64	Female	Unemployed
Parent 4 (Asanda)	28	Female	Unemployed
Parent 5 (Abuze)	51	Male	Salesman
Parent 6 (Alukho)	54	Male	Unemployed
Parent 7 (Alwande)	57	Female	Unemployed
Parent 8 (Akhona)	38	Female	Unemployed
Parent 9 (Azola)	30	Female	Unemployed
Parent 10 (Azande)	48	Male	Intellectual Disability Grant Holder

**Table 2.** Participants’ profile: Inclusive school two.

Participant (Pseudonym)	Age	Gender	Occupation
Parent 1 (Bono)	33	Female	Pre-school principal
Parent 2 (Buhle)	40	Female	Call centre Agent
Parent 3 (Beatrice)	29	Female	Accounts Assistant
Parent 4 (Banele)	39	Male	Technician
Parent 5 (Bonolo)	41	Female	Unemployed
Parent 6 (Bandile)	62	Male	Retired Salesman
Parent 7 (Bontle)	31	Female	Call Centre Assistant Manager
Parent 8 (Busi)	54	Female	Unemployed
Parent 9 (Buyi)	29	Female	Unemployed
Parent 10 (Bobo)	30	Female	Chef

**Table 3.** Participants’ profile: Inclusive school three.

Participant (Pseudonym)	Age	Gender	Occupation
Parent 1 (Carol)	41	Female	Receptionist
Parent 2 (Chuma)	45	Female	Full-time Pastor
Parent 3 (Coliwe)	42	Female	General Assistant

Ethical clearance was obtained from the Ethics Committee of the institution housing this study. Also, research approval was obtained from the Gauteng Department of Education (GDE) to gain entry at the three selected inclusive schools within the Gauteng province. The participants were informed about the aims of the study and given an opportunity to indicate availability to participate. After taking them through the consent form which covered all the ethical considerations, they signed informed consent which assured them of confidentiality, identity protection through the use of verbatim responses on the report (Fleming and Zegwaard, 2018), protection from harm, and the right to withdraw from participation at any point were adhered to within the field of research (Babbie and Mouton, 2008). In addition, the trustworthiness of the study was ensured through the audio-recording of interviews, to which the participants gave permission, as cited by Korstjens and Moser (2018), Mayan (2016) and Elo and Kyngas (2008).

### Data collection methods

Semi-structured interview data collection tool was used to collect data and gain insight into unfolding opinions, experiences, values, and various other aspects of the population under study (Khanzode, 2011; Showkat, 2017). Interviews are optimal for collecting data on individuals’ personal histories, perspectives, and experiences, particularly when sensitive topics are investigated, and to understand how participants construct meanings (McMillan and Schumacher, 2010; Rubin, 2011). Since the study sought to explore how parents construct meanings to their lived experiences, the method was deemed suitable. All interview sessions were 45 minutes long and conducted at the school premises in a private space that was arranged with the school management. Each participant was interviewed once, and contact details were captured to address issues that could require follow-ups at a later stage.

### Data analysis

This study utilized an inductive thematic analysis approach to analyze the interview transcripts and the field notes. The intention was to identify patterns in the data using thematic codes. Utilizing an inductive analysis approach means that the researcher obtains patterns, themes, and categories from data collected, instead of setting and imposing those prior to data collection (Samuels, 2019). Data collection included interaction with the participants and listening and transcribing interviews at the end of each day and comparing those with the field notes to ensure accurate information capturing. Interview data were transcribed verbatim into Word documents for easy management. Although there is no single prescribed method of working with data, Smith et al. (2009) make use of six steps, which were followed as guidelines to make sense of the participants’ experiences. In addition, room for flexibility and innovation despite these clear guidelines and steps for conducting the research (Todorova, 2011). The six steps included reading and re-reading the transcribed data (Khanzode, 2011), initial note-making to assemble the data (Smith et al. 2009), developing emergent themes (Saldana, 2013), searching for connections across emergent themes, moving to the next case to repeat steps 1-4, and looking for patterns across the cases (Smith et al. 2009). When the parents had expressed their experiences and the data transcribed, the themes, patterns, and categories emerged to suggest that the misconception of the mild intellectual disability concept was the main hindrance to the process of home-based learning support provision. The themes carried more weight as they came from the participants instead of being imposed on them (Mayan, 2016: 87; Samuels, 2019).

## Results and discussion

While the four themes that emerged from the data analysis are certainly inexhaustive of what the parents attributed the mild intellectual disability concept to, these themes captured almost all the respondents’ commonalities to indicate parental resistance to the diagnosis and the misconception of the mild intellectual disabilities concept, in the absence of physical disabilities. These themes were: perception of ‘normality’ due to no visible disability, the belief that children had difficulties in reading and writing rather than mild intellectual disabilities, children only struggled with memory, which does not confirm mild intellectual disabilities, children displayed socio-behavioral challenges rather than mild intellectual disabilities. To accentuate their resistance towards the diagnosis and the misconception of the mild intellectual disability concept, parents focused on the dominant presenting symptoms to give more insight into their experiences when providing home-based learning support to their children. Excerpts from data are used in the next section to highlight the misconception of the mild intellectual disability concept as a hindrance to learning support provision by parents. The discussion is supported with literature and the participants’ pseudonyms are used to protect their identity.

## Discussion of findings

### Theme 1: Children are ‘Normal’ in the absence of physical disability

When asked what they understood by the concept of mild intellectual disability, parents indicated their confusion. A common view, which was emphasized amongst all 23 parents was that their children are gifted in some areas of their lives and that this giftedness led to almost all 23 of them finding it hard to accept the use of the term mild intellectual disability to describe the challenges faced by their children. The participants’ overall responses in this study suggested that parents associated the mild intellectual disability concept with physical disability and that the absence of visible physical disability naturally indicated the ‘normality’ of their children. Also, parents’ responses indicated that according to them, their children reached developmental milestones as expected, based on the culture and context within which they existed. In addition, parents indicated confusion that concerns about scholastic performance were never raised at the nursery schools of their children and that only the formal schooling teachers raised concerns. This overall perception of ‘normality’ by parents, due to the absence of visible physical disability concurs with what is alluded to by Donald et al. (2014) and APA (2022) that the onset of mild intellectual disabilities may be unnoticed until the children reach the school-going age.

Andile a parent from the first inclusive school said:

“I do not understand what a mild intellectual disability means, like . . . what is a disability? Does it not mean that my child cannot walk or talk properly? Or perhaps with one hand shorter than the other? Is that not what a disability is?” ^1^

Echoing Andile, Azola said: *“I don't understand what’s going on because my daughter can do the right things at home, I find it hard to accept that you describe her as disabled.”*

In the same breath, Azola rejected the use of the concept of mild intellectual disability when he further said:

“That’s why I don’t want to call it a disability because my daughter can clean, she enjoys cooking, and watching TV and she is neat. She asks questions when she doesn’t understand, and she watches things and understands on her own. The problem is only at school.”

Ayanda also indicated her belief that her son is ‘normal’ when she said: *“He enjoys sitting at home, eating nice food like any other normal person. Why do you describe him as disabled?”*

Echoing Ayanda, Andile’s response was,

“I do not understand what it means that my daughter presents with a mild intellectual disability, like what is a disability? Because she is a normal human being and does normal things like you and I.”

Rejecting the concept of disability Azande said:

“I wouldn’t say it’s intellectual disability, but rather a difficulty that causes them to take long to learn at school.”

Agreeing with Azande, Bandile said,

“My son is not a lazy person, but I become confused when he sleeps when he is doing his work. He is good in arts, and I am not sure if disabled people are able to do art.”

Bonolo also explained her child’s challenges as nothing more than her son’s *“difficulties in the learning environment. Although, he is fine in all the other areas and shows no disability at all.”*

Concurring with Bonolo’s statement, Bontle stated:

“*It’s surprising though that you would call my child disabled because with her phone and singing along to music, she does it very well and remembers all her words…… But school, yoh!”*

Coliwe’s response was in accord with Bontle’s statement when she said:

“I would not say that my son is disabled because he loves doing his homework but just finds it hard. I think the problem is how this new syllabus is structured”

Bobo suggested that educators need to be more patient when he said:

“I also feel that the teachers do not give him enough attention. He would be fine if they did because he is not stupid.”

Carol’s response echoed Bobo’s when she said:

“My daughter just needs someone who is gentle and soft and willing to praise her. ... She is not at all disabled.”

The excerpts of the responses above suggest that the mild intellectual disability concept, for most participants, is associated with a physical or visible disability, without which the parents perceived that their children are ‘normal’. These parents were not acceptive of the use of the term ‘disability’ to describe their children’s scholastic challenges. Although more than three-quarters of the parents saw their children as ‘normal’, only Bandile, one out of 23 parents explicitly added a negative connotation to describe their perception of the term mild intellectual disability during an interview when he said,

“At the moment I know nothing about mild disability. All I know is that a disabled person is someone who is crippled or something like that.”

The overall responses further confirmed findings by Brown (2013), that parents of children with intellectual disabilities often reject the diagnosis initially and that when they finally do, they enter a grieving state because, to them, the diagnosis is equated to a loss of dreams about the bright future for their children. Furthermore, according to Shyman (2016), parents of children with mild intellectual disabilities tend to perceive their children as ‘normal’ and typically developing. This often leads to resistance to the diagnosis. These parents often point out the absence of delays in developmental milestones to support their confusion and rejection of the diagnosis (Centre for Disease Control & Prevention, 2019). Despite the literature which suggests that some parents often miss the red flags in developmental milestones delays, due to children spending more time with their carers while the parents pursue their careers. Laughlin (2013) and APA (2022) assert that the onset of mild intellectual disability symptoms can be observed once the children reach their school-going age.

Based on the parental limited understanding of the mild intellectual disability concept and the perception that their children are ‘normal’, Broberg (2011) concludes that this perception is often associated with denial of the existence of intellectual disabilities. These findings confirm the findings of this study, where on their quest to describe their children with mild intellectual disabilities as ‘normal’, parents highlighted the abilities and strengths of their children and only mentioned the most obvious challenges experienced.

### Theme 2: Children have difficulties in reading and writing rather than mild intellectual disabilities

Disallowing the use of the concept of mild intellectual disability due to the perceived connotations and stigma attached to it, the participants took their explanations further to attribute the challenges experienced by their children simply to challenges with reading and writing. Some felt that their children were misunderstood and not given enough support to address the learning challenges at school. While almost all 23 parents preferred not to use the term mild intellectual disabilities, 11 out of 23 parents across all three schools as per excerpts below, linked the intellectual disability to challenges with reading and writing. The excerpts below were taken from their responses.

Azola said:

“My granddaughter is not stupid, and she speaks English well. There is just a problem with reading and writing.”

Asanda echoed Azola and said:

“I don’t know what this is even, but according to my understanding, it means that the children with mild intellectual disabilities have difficulties in learning, especially with reading and writing.”

Ayanda, on the other hand, supported the two responses when she said:

“I understand that he can’t write and read.”

Additionally, Abuze said:

“*My understanding is that there are barriers to reading, writing, and understanding of languages that are not their native.”*

Bonolo supported the idea of reading and writing challenges when she said:

“My son is not a lazy person, but he surprisingly sleeps when he is doing his work. He is good in arts but struggles with reading and writing.”

Echoing the participants who associated intellectual disabilities with challenges in reading and writing, Ayanda also said:

“I understand that he can’t write and read.”

Andile resonated with Ayanda when he said:

“My child tries, but still struggles, especially with her spelling, reading, and writing, and confuses her letters such as ‘b’ and ‘d’.”

Azola echoed most of the parents by saying:

“... and she speaks English well. There is just a problem with writing.”

Abuze also mentioned that her child had *“barriers to reading, writing, and understanding of languages.”*

Asanda shed more light on the challenge when she said:

“I also write for him because he does not know how to write.”

Alukho sounded hopeful when he said:

“He had a problem in Grade 1 and could not grip his pencil.”

In the same breath, Bono, who had been observing improvement in her child, stated,

“He used to be frustrated as he didn’t understand why he couldn’t read and write.”

While agreeing with all the responses, Buyi felt the need to explain the positives about her son when she said:

“He is clever but academically, no!. He just cannot read but that does not make him disabled at all.”

In summary, parents expressed that they had observed their children’s difficulties with reading and writing. The above responses given by the parents suggested that besides their lack of understanding of the mild intellectual disability concept, they did not feel comfortable with the term. Parents attempted to convince the researcher to see the disability from their perspective, which is that their children simply struggled with reading and writing. The observation of challenges with reading and writing concurred with the mild intellectual disabilities description in the APA (2022), that school-going children with mild intellectual disabilities have difficulties with reading and writing.

Difficulties in reading and writing fall within the conceptual domain that is affected in the presence of mild intellectual disabilities, which include skills in language, reading, writing, math, reasoning, knowledge, and both short-term and working memory (DSM-5, 2013; APA, 2022). Parents may pick such challenges up when providing learning support at home. This information suggested that in their efforts to understand what mild intellectual disability is, parents mostly focused on the outcome of their children’s learning when their children tried to share what they had learned.

### Theme 3: Children only struggled with memory, which does not confirm mild intellectual disabilities

Another sub-theme that emerged from the responses of parents was that they observed poor memory in their children. It was clear that although parents do not fully understand the mild intellectual disability concept, they were aware that the disability affects memory ability. Parents felt that their children were forgetful. The following excerpts from the individual interviews supported this parental perception.

While Alwande stated:

“I think this means that my child has a mental disorder ... He just cannot remember his schoolwork.”

Akhona said,

“All I understand is that there is something wrong in the brain.”

Buhle, on the other hand, showed more insight and alluded to the challenges in memory when she said:

“In Grade 1, the teacher told me that my child only remembers what was taught in that particular day. If you teach him words today and give him a test today, he can get a total. The next day, it’s tough; he remembers nothing at all.”

In support of the responses about mild intellectual disabilities having to do with memory impairments, Carol said:

“My understanding is that the child is struggling to remember things. You can teach your child and you think that she knows everything. Days later when you go back to revision, you realize that they cannot remember anything. It’s like her mind gets stuck. I do not know what happens between learning and later remembering.”

The overall responses in this section strongly suggested that most parents tend to focus only on the dominant challenge faced by their children when sharing their understanding of the concept of mild intellectual disabilities.

According to Saeed and Tahir (2016), children with mild intellectual disabilities experience challenges with visual-spatial and verbal short-term memory. Experiencing challenges with short-term memory implies that the new concepts taught do not register at a similar pace to their typically developing peers. As a result, children miss out on the work covered in class on a normal day. Poor memory later manifests when children with mild intellectual disabilities struggle to remember most of the work covered. Moreover, the parents linking mild intellectual disabilities to memory challenges suggested they have some level of understanding of mild intellectual disabilities, but not sufficient to enhance the provision of adequate support with schoolwork. Parents overlooked the possibility that their children struggle to learn and remember because of reduced cognitive domain ability, as per the definition of mild intellectual disabilities according to the DSM-5 (2013) & APA (2022).

### Theme 4: Children displayed socio-behavioural challenges rather than mild intellectual disabilities

The misconception of the mild intellectual disabilities concept resulted in parents rather limiting the description to the observable behavioural difficulties, which are evidence of the social domain impairments as a source. Asked to share their understanding of mild intellectual disabilities, the overall responses by parents suggested that they associate their children’s scholastic difficulties with the inability to establish healthy relationships with educators and peers at school. As an example of this perception, Ayanda said: *“My son is aggressive. When you question one small thing with him, he fights a lot and he also steals.”*

Shedding more light on the perceived difficulties, Coliwe said:

“My child is short-tempered. I don’t know how to perfectly describe this. My child has friends but is very short-tempered and ends up alone most of the time.”

Azande also shared her anxieties and said:

“I always don’t know what to expect, when to expect and how to expect it . . ‘cause he is angry all the time. Sometimes, he gives me this fear, as I am the only one who can calm him down?”

Echoing Azande, Buhle said:

“If we spend more than 10 minutes on a task, he gets really frustrated and angry and he would rather go out and play.”

These responses indicate that parents perceive that mild intellectual disabilities result in their children not being able to relate well with their peers and teachers, which confirms findings by Donald et al. (2014), that children with intellectual disabilities in general, struggle to form and maintain relationships. This inability to relate was linked by parents to the overall process of teaching and learning being impeded. The overall responses highlighted the lack of mild intellectual disabilities concept understanding by parents and suggested the perceptions of parents in describing the day-to-day challenges faced by their children diagnosed with mild intellectual disabilities.

Donald et al. (2014) asserted that children with mild intellectual disabilities are usually present with secondary problems such as a negative sense of self-worth, low frustration tolerance, and behavioural problems. Also, the parents’ responses suggested that they believed mild intellectual disabilities to be an extrinsic factor, one that results from unhealthy relations between their children and the educators. This perception appeared to hinder parents from fully understanding the concept and their role in providing home-based learning support. Although the parents perceive mild intellectual disabilities to be smaller aspects of the disability as supported by current literature, the prevalence of misconception suggests a need for intervention to raise awareness of the mild intellectual disability concept.

This study sought to address the question about the experiences of parents in supporting their children diagnosed with mild intellectual disabilities in inclusive school settings, with the aim to establish the hindrances to learning support in the home setting. The interest in researching ways to improve learning support results from the promulgation of Education White Paper 6 on inclusive education, as part of promoting access to education for South African citizens, regardless of their abilities (DOE, 2014). This study locates itself within that movement towards strengthening inclusivity by researching hindrances when providing learning support to children with mild intellectual disabilities. The overall findings suggested that parents were not comfortable with the use of the ‘disability’ term in the absence of physical disabilities. In addition to rejecting this term, parents added the negative connotations and stigma associated with the term ‘disability’ in general. Furthermore, all the participating parents’ responses indicated that none of the parents believed that the challenges faced by their children could be linked to the mild intellectual disability diagnosis. Instead, participating parents preferred to highlight specific challenges in isolation and labelled their children as typically developing as they conform to what Shyman (2016) describes as ‘normal’, due to no visible disabilities.

This research is located within the microsystem level of Bronfebrenner’s bio-ecological systems theory, the framework that underpinned the study. This implies that the data was gathered from the immediate and most influential level of the theory, comprising the interactions of the children with their parents and school. The findings revealed that parents (the study participants) have misconceptions about the mild intellectual disability concept, which hinders home-based learning support. In response to this finding, the focus should be placed on empowering the parents as the facilitators of home-based learning support.

In summary, the overall findings concurred with the literature, in that most parents perceive their children with mild intellectual disabilities as ‘normal’ or typically developing (Shyman, 2016). Furthermore, parents alluded to how the use of mild intellectual disabilities concept brings discomfort to them as they often do not agree with their children’s diagnosis. Such a finding is in contrast with what the study by Blacher and Baker (2007) uncovered, which suggested that intellectual disability diagnosis has a positive impact on families as they often hold hands to provide learning support to their child who receives the diagnosis. Additionally, this study's findings contrasted those from a systematic review conducted by McGarty and Melville (2018) on parental perceptions of facilitators and barriers for children with intellectual disabilities. The review revealed that most parents often seek innovative ways to convert barriers in providing support to children with intellectual disabilities, into enablers of learning support facilitation.

In this study, parents displayed misconceptions of mild intellectual disabilities concept by rejecting the use of the term ‘intellectual disabilities' and suggesting their preference to discuss the symptoms they observed, such as challenges in reading and writing, confirmed by the Centre for Disease Control and Prevention (2019), poor memory, as cited by Saeed and Tahir (2016), and behavioural challenges, cited by Donald et al. (2014). Moreover, this study's findings suggest that South African parents lack clarity about the concept of mild intellectual disabilities. Ultimately, this study unmasked the misconception of the mild intellectual disabilities concept as the main hindrance to the process of providing home-based learning support for children with mild intellectual disabilities (Statistics South Africa, 2016).

## Recommendations

The results of this study confirmed literature that there is currently a dearth of literature published on educating parents about intellectual disabilities and providing strategies to empower parents, who are primary educational stakeholders with the necessary skills to support children with mild intellectual disabilities (Dunne, 2015). Therefore, this study recommends psycho-education for the parents by the Department of Education at the district level. This would be spear-headed by the district-based support team (DBST) through the inclusion of parental workshops as part of their annual plan to service the schools that fall within the district office’s jurisdiction. According to Visser and Moleko (2019), an impactful dissemination of knowledge is one that focuses on providing systematic information on the phenomenon at hand. The understanding of and the ability to support children with mild intellectual disabilities is the phenomenon at hand in this case. In addition, parental empowerment could come when available treatment options, the educational needs of those diagnosed, and strategies for families to cope better are adequately covered in the interventions. Coping strategies should also unpack tips on how to respond to the educational needs of individual children with mild intellectual disabilities. Epstein and Sheldon (2019) allude to the best practice in knowledge distribution as an intervention or a guideline which begins with the investigation of the level of phenomenon understanding. Based on the alluded best practice guidelines, the DBST would need to establish the level of intellectual disabilities knowledge by parents and develop psycho-educational interventions to suit the parents’ needs. It is important to note that the literature suggests that such interventions at the community level require the utilization of assets that are already available within the community (Caño et al., 2016). Involving the community, particularly parents, is in line with Bronfenbrenner’s bio-ecological systems theory which underpinned this study. This theory emphasizes the importance of considering the context within which the participants exist for impactful and sustainable interventions. Using Bronfebrenner’s bio-ecological systems theory to guide the intervention is envisaged to impact the policy and practice of the stakeholders involved.

## Limitations

The sample size poses a limitation to the study as only 23 parents of children with mild intellectual disabilities from one province participated. This limits the generalization of the misconception of mild intellectual disabilities concept by parents from other provinces within South Africa. The second limitation was the method used for data collection which was semi-structured interviews. This could be limiting in that the study relied on a single method of data collection, whereas the use of various data collection methods could have provided different perspectives. Nevertheless, this method obtained participants’ lived experiences, which provided a rich understanding of the hindrances to the provision of home-based learning support by parents. Lastly, there was a missed opportunity to explore whether there were any cultural differences in the perspectives of the mild intellectual disability phenomenon. However, this highlighted the need for further research in the area.

## Conclusion

Using the semi-structured interviews, this study investigated the experiences of parents in providing home-based support to their children diagnosed with mild intellectual disabilities, to unmask the hindrances to learning support provision. The overall finding suggested that parents have misconceptions about the mild intellectual disability concept. Their responses were classified under four themes, which were: perception of ‘normality’ in the absence of physical disability, the belief that children had difficulties in reading and writing rather than mild intellectual disabilities, children only struggled with memory, which does not confirm mild intellectual disabilities, children struggled with socio-behavioural challenges rather than mild intellectual disabilities. In addition to highlighting the misconceptions about the mild intellectual disability concept, the misconception was highlighted as a hindrance to the provision of learning support in the home setting. As a result, a recommendation was made to focus on parental empowerment to place parents in a better position to provide learning support to their children with mild intellectual disabilities.

## Data Availability

Data sharing does not apply to this article as no new data were created or analysed in this article. Data from the D.Ed study, accessible from the University of Johannesburg library repository were used in the writing of this article.

## Note

1. The pseudonyms were used to extract excerpts from the main study responses.
